# How bilingualism modulates selective attention in children

**DOI:** 10.1038/s41598-022-09989-x

**Published:** 2022-04-16

**Authors:** Jacqueline Phelps, Adam Attaheri, Mirjana Bozic

**Affiliations:** Department of Psychology, University of Cambridge, Downing Street, Cambridge, CB2 3EB UK

**Keywords:** Neuroscience, Psychology

## Abstract

There is substantial evidence that learning and using multiple languages modulates selective attention in children. The current study investigated the mechanisms that drive this modification. Specifically, we asked whether the need for constant management of competing languages in bilinguals increases attentional capacity, or draws on the available resources such that they need to be economised to support optimal task performance. Monolingual and bilingual children aged 7–12 attended to a narrative presented in one ear, while ignoring different types of interference in the other ear. We used EEG to capture the neural encoding of attended and unattended speech envelopes, and assess how well they can be reconstructed from the responses of the neuronal populations that encode them. Despite equivalent behavioral performance, monolingual and bilingual children encoded attended speech differently, with the pattern of encoding across conditions in bilinguals suggesting a redistribution of the available attentional capacity, rather than its enhancement.

## Introduction

The maturation of selective attention is arguably one of the key developmental processes, with even subtle modifications to this process potentially leading to significant consequences. This reflects the findings that selective attention is not only linked to inhibitory control^1^ and working memory^2^, but associated with the development of a variety of skills including speech^3^, metalinguistic skills^4^ and arithmetic^5^. In fact, selective attention is proposed to be one of the key foundational skills for academic success in children overall^6,7^.

One of the factors that has been linked to modification of selective attention in children is bilingualism. Learning and using multiple languages is a major processing demand for the cognitive system, with evidence showing that bilingual language use leads to parallel activation and competition between the two languages, requiring the users to selectively prioritise one and inhibit the other^8–10^. Our neurocognitive system accommodates these additional processing demands by modifying and adapting the underlying neural and functional architecture, as evidenced by a large number of studies in both children and adults^11–13^. Importantly, effects of bilingualism on aspects of neurocognitive processing have been observed from very early on, with data showing differences between monolingual and bilingual infants as young as 4–6 months old^14,15^ as well as in older children^16,17^.

Yet, the nature of these adaptive changes is still not entirely clear. One widely held view is that the increased processing demands arising from bilingual language use lead to enhanced capacity for selective attention, resulting in better performance for bilingual children on selective attention tasks^18,19^. However this view has also been challenged, with a number of reports either not finding evidence for enhanced performance in bilinguals^20–22^ or arguing that they can be accounted for by variables other than bilingual experience^23,24^. One notable finding relevant in this context is that neural differences between monolinguals and bilinguals have been observed even when they display equivalent behavioural performance^25–27^ suggesting that these neuroadaptive changes might be explained by a different mechanism instead. For instance, a recent paper^27^ compared behavioural performance and the neural encoding of attended and unattended spoken narratives in monolingual and bilingual speakers. Participants were instructed to listen to a story in their native language, while ignoring different types of linguistic and nonlinguistic interference presented in the other ear. The results showed that, even though the respondents’ comprehension scores were the same, there were significant differences in the pattern of neural encoding of the attended streams between the monolingual and the bilingual group.

With this in mind, a somewhat different interpretation of the mechanisms of neurocognitive adaptations in bilingualism might be that they emerge in order to enable bilingual children (and adults) to achieve and maintain optimal behavioural performance under the increased processing demands of bilingualism. Importantly, this compensation for the more complex processing environment is achieved in the context of a finite selective attention capacity. This account acknowledges that attention is ultimately limited such that we can only process a restricted amount of information at any given point^28,29^, and that selective attention might also require processing capacity itself^30,31^. In the bilingual context this could mean that the process of selecting the target language and inhibiting the non-target one will itself utilise some of the existing attentional resources. This would then impact on the remaining attentional resources such that they need to be economised in order to support optimal task completion. This view builds on, and extends, the hypothesis that bilingual control processes themselves adapt to the recurrent processing demands placed upon them (the adaptive control hypothesis^32,33^), and while it does not preclude the possibility that this may lead to greater flexibility in the usage of the residual capacity, it shifts the focus from the often-inconsistent behavioural comparisons to the patterns of modification and adaptation in the underlying neural and functional architecture. Critically however, this account also gives rise to a different set of predictions about the patterns of these underlying adaptations. In particular, instead of assuming an overall enhancement in neural indices of attentional processing for bilinguals compared to monolinguals, this view predicts no increase—or possibly even a slight reduction—combined with their different distribution as determined by the requirements of the task at hand.

### Current study

To dissociate between these alternatives and establish how bilingualism modulates attentional processing in children, the current study investigated the neural encoding of attended and unattended speech envelopes in monolingual and bilingual listeners aged 7–12. The neural encoding of speech envelopes is a well-established method for investigating attentional processing, which builds on findings that attention causes low-frequency neural oscillations to entrain to the temporal envelope of speech (‘selective entrainment hypothesis’^34,35^). There is a large body of evidence confirming robust correlation between attended speech envelopes and neural activity^36–39^ and showing that the neural encoding of speech envelopes plays an important role for speech intelligibility in both adults^40^ and children^41,42^. The current study thus employed EEG to capture the neural encoding of attended speech envelopes in monolingual and bilingual children. We used linear regression as implemented in the mTRF toolbox^43^ to model the relationship between the speech signal and the neural data, and applied it in a backward direction to assess how well the attended and unattended speech envelopes could be reconstructed from the responses of the neuronal populations that encode them (see “Materials and methods” section for more details). The accuracy of speech envelope reconstructions from the EEG data was assessed by comparing the reconstructions to the original speech envelopes, resulting in reconstruction accuracy scores (Pearson’s r)—where the higher r value signifies that more stimulus-relevant information was encoded in the EEG signal and the better model could be created, leading to a better reconstruction. Reconstruction scores calculated this way are widely accepted as measures of neural encoding in children^41,44^ and are consistent with other computations of cortical tracking^45^. Another feature of the reconstruction method is that it maps all available neural data simultaneously and is therefore specifically suited to multi-channel systems such as EEG. The mTRF technique has also been shown to be particularly suitable for natural speech^37,46^.

Another important consideration for investigation into the ways bilingualism shapes selective attention in children is the trajectory of selective attention development. Auditory selective attention is proposed to have developed by age 3–5^6^ and auditory dichotic tasks have been carried out on children as young as 4^7^. Yet a minimum age of 6 has been recommended^47^, reflecting the inconsistent results and high variance in response speed and accuracy in the younger children^48^. In addition, the established view is that selective attention only stabilises around the age of 7^49^ and reaches maturity by the age 8 or 9^50^. Given these considerations, in the current study we recruited participants in the age range of 7–12, as this age range not only represents a developmental plateau for selective attention in childhood, but is also likely to generate relatively stable effects whilst ensuring that children can reliably perform a selective attention task.

To investigate whether and how bilingualism modifies the neural mechanisms of selective attention in children, the current experiment used a dichotic listening task^51^. Following the design we used previously^27^, children were presented with two competing narratives simultaneously and instructed to attend to one while ignoring the other. The nature of the competing stream was manipulated across four different conditions to create perceptual or linguistic interference. The first condition was ‘Single talker’, a control condition where children attended to a narrative presented in one ear, with no interference presented in the other ear. This allowed us to establish the extent of attentional encoding in monolingual and bilingual listeners at baseline (i.e., without any interference present). In the second condition, children attended to a narrative in English presented in one ear while ignoring another English story presented in the other ear (English–English condition). In the third condition, children attended to a narrative in English while ignoring a narrative in Latin, a language unknown to them (English–Latin condition). These two conditions therefore tested attentional encoding in the context of linguistic interference, where the known language distractor (English) could be expected to interfere more strongly with the attended stream than the language that children cannot process for meaning (Latin). In the fourth condition, the interfering stream was Musical Rain (MuR), a nonlinguistic sound that is closely matched to the acoustic properties of speech, but does not trigger speech percept and is therefore expected to only engage low-level acoustic processing (English–MuR condition). Another key feature of this design was that participants were instructed to listen to the attended stream for comprehension, a task that we expected that children in this age group would be able to do without difficultly. Based on the existing adult data^27^ we also expected that there would be no significant difference between the ability of monolingual and bilingual listeners to perform the task. By equating on behavioural performance, this approach enabled us to focus on the patterns of modification of the mechanisms that underpin selective attention, rather than performance per se.

The set of conditions described above allowed us to investigate whether bilingualism modifies the neural underpinnings of selective attention in children, and to directly assess the predictions of the two hypothesised mechanisms of this modification discussed earlier. Following the existing evidence^27,37–39^, we assumed that attention would modulate the neural encoding of speech envelope in both monolingual and bilingual children, with the type of distractor probably further influencing the strength of the encoding of the attended stream. Critically however, we assumed that the way different distractors influence attentional encoding might differ between the groups. According to the hypothesis that bilingual experience leads to general enhancement of attentional processing, we would expect to see an overall increase in reconstruction accuracy scores for bilingual compared to monolingual children. Specifically, while the overall *pattern* of effects might be similar in the two groups—with linguistic distractors likely causing stronger interference than the non-linguistic distractor and the Single talker condition—all these markers of attentional encoding would be expected to be enhanced in the bilingual group. On the other hand however, we might observe no increase, or even a decrease in the indices of attentional encoding in bilingual children, reflecting the hypothesis that language selection and inhibition themselves might draw on the existing attentional capacity, restricting the resources available to track the speech envelope. In addition and more importantly, this could lead to a modification of the encoding patterns across conditions in bilinguals, suggesting that the remaining attentional capacity has been distributed to maximise this finite resource and meet the task requirements in the context of increased processing demands of bilingualism.

## Materials and methods

### Participants

48 typically-developing children aged 7–12 were tested, comparable to the sample size of similar EEG studies on children^37,41,44,52^. They were split into two categories: bilingual (n = 24, sixteen males, age M = 9.3 year, SD = 1.83) and monolingual (n = 24, thirteen males, age M = 9.6 year, SD = 1.48), which were matched groupwise on mean and distribution of age (t = 0.54, *p* = 0.59). All participants were healthy with no history of hearing problems or neurological disorder. 43 were right-handed, with four of the left-handed children being monolingual and one bilingual. All participants’ parents completed a language history questionnaire, which provided an overview of children’s exposure to languages. As confirmed by the questionnaire, all monolingual participants were native speakers of English, with no significant exposure to other languages. The participants in the bilingual group all had a similar profile: the language they first learnt was not English, and they used this language at home on a daily basis. They were however fluent and highly proficient in English, following English-speaking curriculum at school, and with native-like English conversation skills comparable to their monolingual peers. The second languages spoken were Afrikaans, French, Finnish, Greek, Hindi, Hungarian, Igbo, Japanese, Lithuanian, Mandarin, Polish and Turkish. Additionally, two children spoke a third language proficiently (French and Spanish), and one spoke a total of four languages other than English proficiently (Arabic, French, Hebrew and Spanish). Children were recruited via posters, social media, and word of mouth. Parental education information was collected as an indication of SES, a well-documented influence on selective attention in children^52^. The majority of participants’ parents (87.2%) were educated to degree level or higher, and the groups were not significantly different on this approximation of SES (bilinguals M = 2.56, SD = 0.52; monolinguals M = 2.35, SD = 0.79; Mann–Whitney U = 259, *p* = 0.53).

The study was approved by the Cambridge Psychology Research Ethics Committee, and performed in accordance with relevant guidelines and regulations. Prior to the testing session, parents and children were given detailed information on the aims of the project and what to expect from the session. Upon arrival, informed consent was given by parents signing a consent form and the children an assent form. They were told they could withdraw from the study at any time.

### Design

The experiment consisted of four conditions (Table 1). In each condition, children were attending to a story in English in one ear. Condition 1 had no interference in the other ear (‘Single talker’). In the other three conditions children were also presented with a distractor in the other ear, which they were instructed to ignore. The nature of the distractor varied, from a different story in English (‘English–English’), to a story in a language unknown to children (‘English–Latin’) and non-linguistic acoustic interference (‘English-Musical Rain’).

**Table 1:** Experimental conditions.

Condition	Attended stream	Interference
1. Single talker	English story 1	No interference
2. English–English	English story 2	Different story in English
3. English–Latin	English story 3	Story in unknown language (Latin)
4. English–MuR	English story 4	Nonlinguistic acoustic interference (Musical Rain)

The target stories for the attended ear were four children’s stories in English specifically aimed at this age group, taken from online resource storynory.com. All stories were transcribed into 120 sentences each, with each sentence lasting approximately 3 s in length. Each target story was then split into 2 blocks and children attended to the first half in either the left or right ear (randomly assigned), with interference in the other, and then swapped ears for Block 2. Each block (half of a story) consisted of 60 sentences, with all 60 sentences concatenated with a 300 ms gap between them to create a single block lasting 3.3 min. Block 1 was always the first half of the target story and Block 2 the second half. Latin was chosen as the interference in Condition 3 as a non-artificial language which would almost certainly be unknown to the participants. Gender of the speaker was kept the same for all stories (same female voice for all target stories, different female voice for interference), to reduce segregation strategies based on talker’s gender^53^. All stories’ volumes were normalised to ensure equivalent average amplitude. The non-linguistic interference of Musical Rain was identical in length, root mean squared level and long‐term spectrotemporal distribution of energy to the target story in Condition 4, but did not trigger a speech percept^54^. It was generated in MATLAB by extracting temporal envelopes of the target sentences and filling them with 10 ms fragments of synthesized vowels jittered in frequency and periodicity. The resulting stream was described by participants as “the sound of a jug pouring water”. Instructions were recorded by the same female speaker of the target stories. These were played before each block in the target ear, telling the child*:* “This is your right/left ear. Please listen carefully to the story in this ear, on your right/left side, and ignore the story or sound in the other ear”.

### Procedure

**Figure 1:**
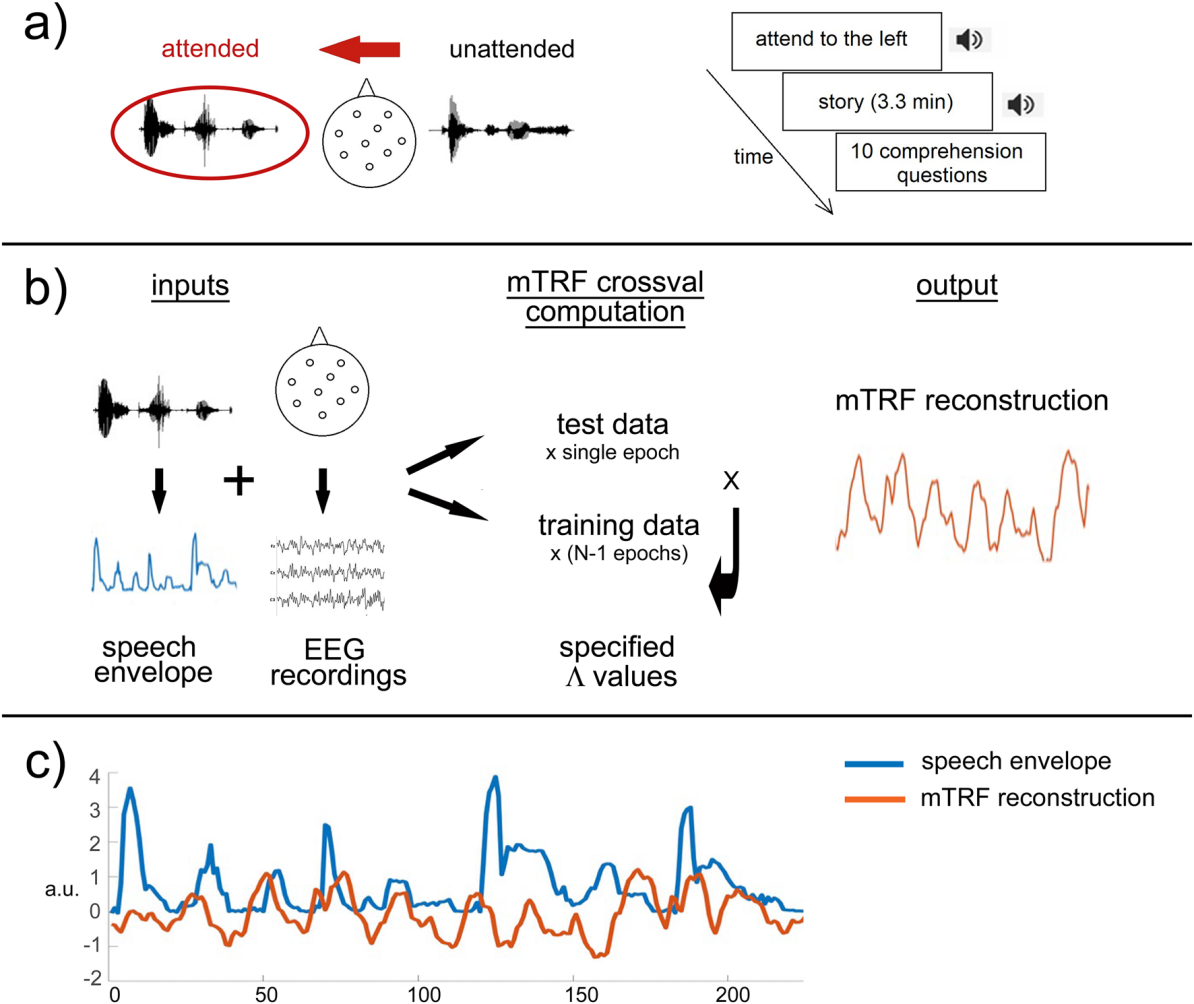
Experimental procedure and mTRF model computation. (**a**) Procedure: Children were instructed to attend to one side. The stimuli were presented for 3.3 min, and children were then asked to complete 10 comprehension questions about the attended story. (**b**) mTRF stimulus reconstruction: A backwards mTRF decoding model was fit separately to the speech envelope of each of trials for each participant, using a leave-one-out cross-validation procedure. This generated a reconstruction of each speech envelope that was validated against the original stimulus envelope. (**c**) Reconstruction accuracy score: The blue line shows the speech envelope from one trial of the original stimulus. The orange line is the estimate of the envelope reconstructed by the decoder. The reconstruction accuracy score (r) is a measure of the correlation between the original (blue) and reconstructed speech envelope (orange). Resulting r values per sentence per condition per participant were used in statistical analyses.

The participants had a practice session of listening to an English story in both the left and the right ear while ignoring a distracting English story in the other ear, in order to familiarise themselves with the dichotic listening paradigm. After practice, they were asked to summarise the target story to check they could hear correctly and understood the instructions to attend to one ear at a time. The task itself took 45–60 min. Children first heard Block 1 of Condition 1 (Single talker) followed by 10 comprehension questions. They then listened to Block 2 of Condition 1 (Single talker), again followed by 10 comprehension questions. Each block was preceded by the recorded instructions in the relevant (target) ear. This procedure was repeated for the other three conditions, which were presented in a random order. Children were instructed to stay as still as possible while the stories were playing and were allowed to stretch, yawn etc. during the comprehension breaks. An example sequence of a block is presented in Fig. 1a. Comprehension questions consisted of simple sentences to check understanding of each story (for example: ‘This story is about a QUEEN/KING’), and children pointed or verbally confirmed which option they thought was correct. The children did not receive feedback on their responses. At the end of the experiment children were presented with a certificate of completion and compensation for their time.

### Data collection and preprocessing

EEG was recorded using a 64-channel electrode net (Electrical Geodesics Inc., Eugene, OR, USA), connected to Netstation software. The stimuli were played through foam-tipped earphones in the pre-allocated part-randomised order. All data were pre-processed in MATLAB: EEGLAB Toolbox^55^. Channels 61–64 (located in muscular/facial areas) were removed, leaving data from 60 channels for processing. Data was filtered between 1 and 100 Hz using zero-phase bandpass Hamming windowed FIR filters (transition band widths of 1 Hz with cutoff frequencies at − 6 dB) and down-sampled to 250 Hz. Bad channels were identified via probability and kurtosis and were interpolated (via spherical interpolation) if they were 5 SD away from the mean kurtosis and 3 SD from the mean power spectrum. Independent Component Analysis (ICA) algorithm (EEGLAB) was conducted to identify components corresponding to artefacts (e.g. eye blinks). These were visually inspected and bad components removed from the data. After ICA, epochs were extracted, starting at 200 ms pre-onset of the sentence and ending at 2800 ms post onset. This length of epoch was chosen so that, after allowing for epoch rejection, there would be a minimum threshold of five minutes of data per condition for input to the mTRF toolbox^56^. After the bad channels were interpolated, bad epochs were rejected with the *pop_autorej* function (EEGLab), removing epochs with values outside a 3SD of the probability and kurtosis thresholds. This resulted in an overall epoch rejection of 16.33% for all participants’ data (18.31% for monolinguals and 14.35% for bilinguals). By condition, epoch rejection was 14.67% in Single talker, 19.68% in English–English, 12.77% in English–Latin and 17.36% in English–MuR datasets. Next, data were re-referenced to the average of all channels and finally resampled to 100 Hz to reduce the computational load. Following this process, the EEG data from all 48 participants were entered into the subsequent mTRF analysis.

### Speech envelopes

Speech envelopes were calculated using the Hilbert2 function in EEGLAB, downsampled to 100 Hz to match the data and normalised using *nt_normcol* (Noisetools http://audition.ens.fr/adc/NoiseTools/).

### Analyses

Neural tracking of the stimulus envelopes was computed using multivariate temporal response functions, as implemented in the mTRF toolbox^43^. TRF uses linear regression to model the relationship between speech input and signal at each EEG channel. We used the backward model (reconstruction), which has the advantage of mapping all available neural data simultaneously across all channels, calibrating their relative influence so that informative channels receive greater weights than those which provide less data, and dividing out any autocovariance between channels. This way, even stimulus features that are not explicitly encoded in the neural response in a one-to-one mapping may be inferred from correlated input features that are encoded, which would not be the case using direct correlation to the raw signal. The inputs to the calculation of the TRF models were the stimulus (normalised speech envelope), response (normalised EEG data), minimum and maximum time lags, sampling rate and a series of ridge regression parameters (λ). To calculate the models, we created matrices of EEG data and matching stimuli for each attended and unattended condition per participant per group. The size of the matrices corresponded to the number of viable epochs per condition (minimum 100 for a single condition in each participant). Decoder weights over time lags from 0 to 250 ms were calculated using the cross validation (mTRFcrossval) function. The cross validation uses a ‘leave-one-out’ computation which first fits individual models to every trial for each specified λ, then excludes one trial at a time (‘test set’) while averaging the others across models (‘training set’). The averaged model from the training set is then convolved with the test set to generate a stimulus reconstruction. In each model, this was done in rotation with each trial serving once as the ‘test set’, repeated across all λ values (12 λ values, 1 × 10^−3^:1 × 10^8^). Each reconstruction was then validated against the original stimulus, resulting in 12 reconstruction accuracy scores (Pearson’s r) per stimulus, with the *r* value at the optimal λ (identified as that which yields the highest overall *r*-value across epochs) taken. This optimal lambda value selection mitigated against the potential overfitting of the TRF model. The reconstruction accuracy scores were then compared across groups, attention status and condition using linear mixed-effect models^57^ as implemented in the lme4 R package^58^. To arrive at the best-fitting model, we used the step function in the lmerTest package^59^. The Satterthwaite approximation^60^ was used for degrees of freedom. Significant *p* values are reported at *p* < 0.05. All post-hoc tests were FDR corrected for multiple comparisons. Figure 1b,c illustrate the procedure of mTRF model computation, and the outcome of reconstruction for a sample sentence ‘This cat was getting skinnier and skinnier’.

## Results

### Behavioural comprehension scores

Children from both groups performed the task equally well, with overall comprehension scores of 98.1% in the monolingual group, and 98.7% in the bilingual group. To test for any differences between them, we converted the comprehension scores to Z scores, then ran a model with Z scores as the dependent variable, and factors of group (two levels: monolingual, bilingual), condition (four levels: Single talker, English–English, English–Latin and English–MuR) and their interaction, in addition to participant age and parental socio-economic status (SES), plus subjects as a random effect. The results showed that the only significant factor was condition [F(3,141) = 9.34, *p* < 0.001, η^2^ = 0.17], with no effect of group nor interaction between condition and group. Post hoc t-tests revealed that this effect of condition was driven by a significant difference in performance between the English–English condition and all the other conditions [Single talker: t = 3.27, *p* < 0.05, *d* = 0.67; English–Latin: t =  − 3.03, *p* < 0.05, *d* =  − 0.62; English–MuR: t =  − 2.62, *p* < 0.05, *d* =  − 0.53]. These results show comparable performance of monolingual and bilingual children in the behavioural comprehension task, with the performance in both groups suffering slightly in the English–English condition compared to the other conditions. This substantiates reports by participants in both groups that they found the interference in English the most difficult. A summary of comprehension scores, standard deviations and their differences between groups are shown in Table 2.

**Table 2:** Comprehension scores and standard deviation by condition and group.

Condition	Monolinguals	Bilinguals	t(46)	p
Single talker	99.6 (1.41)	99.2 (2.41)	0.73	0.62 (ns)
English–English	95.8 (6.02)	96.7 (6.7)	− 0.45	0.65 (ns)
English–Latin	98.8 (2.66)	99.6 (1.41)	− 1.36	0.39 (ns)
English–MuR	98.3 (3.18)	99.4 (2.24)	− 1.31	0.39 (ns)
**Overall across conditions**	**98.1% (3.92)**	**98.7% (3.92)**	**− 1.01**	**0.31 (ns)**

### EEG DATA

#### The effects of attention on speech reconstruction accuracy

In the analysis of the neural data, datapoints more than 1.5 interquartile ranges above the upper quartile or below the lower quartile were removed as outliers, excluding 170 datapoints (0.5% of the total). Visual inspection of residual plots did not reveal any obvious deviations from normality. The first analysis of the neural data aimed to test the robustness of the paradigm, by establishing whether attention modulated speech reconstruction accuracy in children. It included the three conditions where both attended and unattended narratives were presented to the participants (English–English, English–Latin and English–MuR); thus excluding the condition where there was no interference (Single talker). The dependent variable was reconstruction accuracy score (r), and the fixed factors were group (two levels, monolingual, bilingual), attention (two levels, attended and unattended) and condition (three levels), and the interactions between them. We also included participant age and parental SES as predictors, and subjects and items as crossed random effects. Results showed a significant effect of attention [F(1,712.8) = 46.53, *p* < 0.001, η^2^ = 0.06]; a significant effect of condition [F(2, 720.6) = 59.3, *p* < 0.001, η^2^ = 0.14] and a significant interaction between condition and attention [F(2, 710) = 5.4, *p* < 0.01, η^2^ = 0.01] as well as between condition and group [F(2, 28,096.1) = 7.4, p < 0.001, η^2^ = 0.005] Pairwise comparisons confirmed that the attended stream EEG data showed on average higher stimulus reconstruction accuracy than the unattended ones, with the difference between them significant overall [r_attd_ = 0.057, r_unattd_ = 0.040, t = 8.79, *p* < 0.001, *d* = 0.10] and in each condition separately [English–English r_attd_ = 0.047, r_unattd_ = 0.032, t = 4.21, *p* < 0.001, *d* = 0.09; English–Latin r_attd_ = 0.053, r_unattd_ = 0.026, t = 8.39, *p* < 0.001, *d* = 0.17; English–MuR r_attd_ = 0.071, r_unattd_ = 0.062, t = 2.38, *p* < 0.05, d = 0.05]. They confirm that attention improves reconstruction accuracy of spoken narratives in children, replicating similar results in the literature.

**Figure 2:**
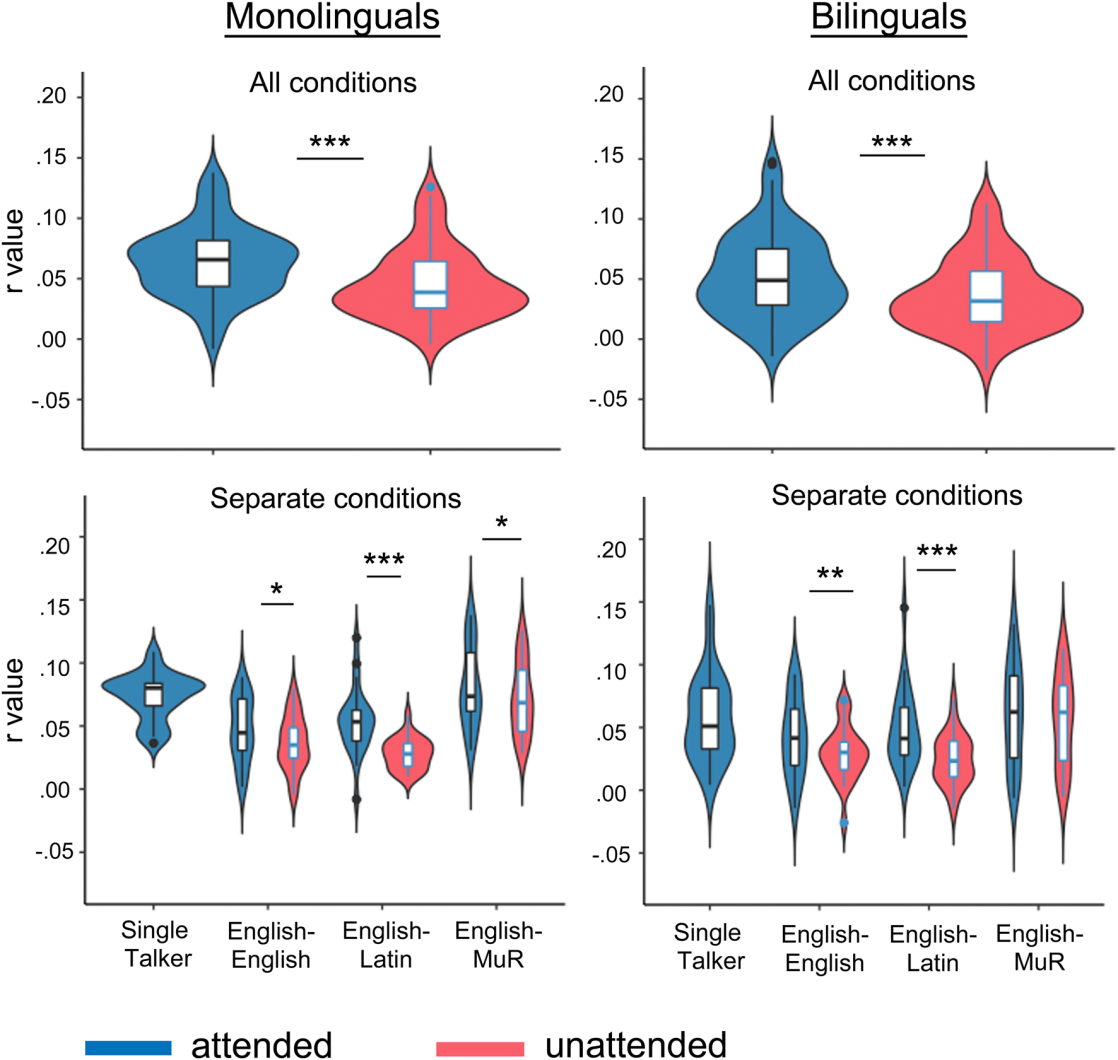
Reconstruction scores for attended and unattended streams per group and condition. Results show robust effects of attention on the reconstruction accuracy of speech envelopes, with higher reconstruction accuracy for the attended than for the unattended envelopes in both groups.

We next tested whether the same general pattern holds in monolingual and bilingual groups separately. In *monolinguals*, a model including attention (two levels, attended and unattended), condition (three levels) and their interaction, participant age, parental SES, plus subjects and items as crossed random effects showed significant effects of attention [F(1, 706.03) = 29.5, *p* < 0.001, η^2^ = 0.04] and condition [F(2, 718.01) = 55.28, *p* < 0.001, η^2^ = 0.13]. In *bilinguals*, the equivalent model showed a significant effect of attention [F(1, 731.19) = 29.03, *p* < 0.001, η^2^ = 0.04], condition [F(2, 731.52) = 21.44, *p* < 0.001, η^2^ = 0.06], and their interaction [F(2, 724.01) = 4.66, *p* < 0.01, η^2^ = 0.01]. Pairwise comparisons confirmed that in both groups attended streams were reconstructed more accurately than unattended streams in each condition separately, other than in the English–MuR condition in bilinguals. Table 3 and Fig. 2 show reconstruction accuracy scores by group and condition.

**Table 3:** Reconstruction accuracy scores (r) by condition and group.

Condition	Monolinguals					Bilinguals				
	attd	unattd	*t*	*p*	*d*	attd	unattd	*t*	*p*	*d*
Single talker	0.075					0.06				
English–English	0.048	0.036	2.59	< 0.05	0.08	0.045	0.029	3.29	< 0.01	0.1
English–Latin	0.055	0.028	5.99	< 0.001	0.17	0.051	0.025	5.87	< 0.001	0.16
English–MuR	0.085	0.073	2.45	< 0.05	0.07	0.059	0.054	0.99	ns	0.03
**Overall across conditions**	**0.066**	**0.045**	**6.36**	**< 0.001**	**0.11**	**0.054**	**0.036**	**6.01**	**< 0.001**	**0.1**

*attd* attended stream, *unattd* unattended stream.

#### Reconstruction accuracy of attended streams in monolinguals and bilinguals

A key question driving this research was to establish whether bilingualism modulates the neural encoding of attended speech envelopes in children; and what pattern does this modulation follow. The next set of analyses therefore asked whether monolingual and bilingual groups differ in reconstruction accuracy of attended streams across conditions. To this end we ran a model that included attended condition (four levels: Single talker, English–English, English–Latin, English–MuR), group (monolingual, bilingual) and their interaction, participant age, parental SES, plus subjects and items as crossed random effects. The results showed that the only significant predictors were condition [F(3, 483.2) = 13.63, *p* < 0.001, η^2^ = 0.08] and group by condition interaction [F(3, 18,283.7) = 3.59, *p* < 0.05, η^2^ = 0.005].

**Figure 3:**
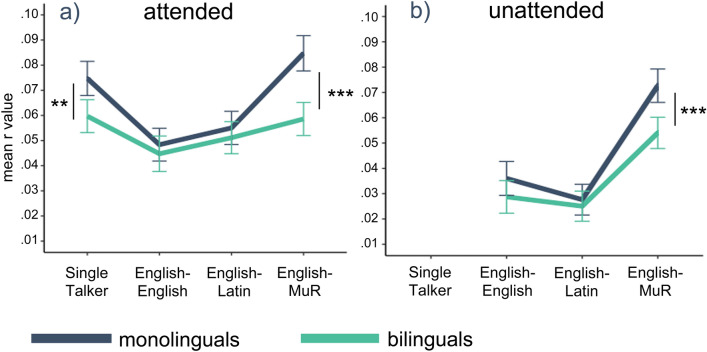
Between-group differences in reconstruction accuracy scores per condition. Summary of the pattern of results for (**a**) attended streams, and (**b**) unattended streams. Error bars represent 95% CI.

To explore what is driving this interaction, we investigated the patterns of reconstruction across attended conditions in each group separately. In *monolinguals*, a model with four levels of attended condition, participant age, parental SES, and subjects and items as random effects, showed a significant effect of condition [F(3,481.02) = 15.1, *p* < 0.001, η^2^ = 0.09] only, with the post-hoc tests showing significantly higher encoding in the Single talker condition than in the English–English and English–Latin conditions [t = 5.49, *p* < 0.001, *d* = 0.16, and t = 4.07, *p* < 0.001, *d* = 0.19 respectively], and significantly higher encoding in the English–MuR than the English–English and English–Latin conditions [t =  − 7.44, *p* < 0.001, *d* =  − 0.22; and t =  − 6.04, *p* < 0.001, *d* =  − 0.18 respectively]. There was a trend of stronger encoding in the Single talker than in the English–MuR condition [t =  − 2.01, *p* = 0.054]; but no difference between the English–English and English–Latin conditions [t =  − 1.41, *p* = 0.16]. The equivalent analysis in *bilinguals* showed a comparable, but much reduced pattern of differences between conditions, with a significant effect of condition [F(3,491.09) = 4.03, *p* < 0.01, η^2^ = 0.02] reflecting weaker encoding in the English–English condition compared to the Single talker and English–MuR conditions [t = 3.05, *p* < 0.05, *d* = 0.09; and t =  − 2.81, *p* < 0.05, *d* =  − 0.08 respectively]. No other differences emerged in the bilingual group, implying that the type of interference significantly modulated attentional encoding in monolinguals but had an attenuated effect in bilinguals, comparable to the results seen in adults^27^. These results are summarised in Fig. 3a.

To confirm that monolinguals indeed encoded attended stream envelopes more strongly than bilinguals in some of the conditions, we directly compared the reconstruction accuracy between the groups in each attended condition separately. These pairwise comparisons for individual conditions showed significantly higher attentional encoding in monolinguals than in bilinguals in the Single talker and English–MuR conditions [t = 3.12, *p* < 0.01, *d* = 0.09; and t = 5.32, *p* < 0.001, *d* = 0.16, respectively], but no difference between the groups in the two linguistic interference conditions [English–English: t = 0.74, *p* = 0.46; English–Latin: t = 0.83, *p* = 0.46]. Hence, even if the attentional encoding in the linguistic interference conditions in bilinguals was comparable to that seen in monolinguals, the significantly weaker encoding of the Single talker and the English–MuR conditions in this group has resulted in the overall much flatter pattern of results across conditions in bilinguals (Fig. 3a). In other words, the key underlying variable behind the differences between the two groups appears to be the strength of attentional encoding in conditions of weak or no interference.

#### Reconstruction accuracy of unattended streams in monolinguals and bilinguals

Following the same approach as used in the analyses of the attended streams, we ran an equivalent model with unattended conditions (three levels: English–English, English–Latin, English–MuR), group (monolingual, bilingual) and their interaction, participant age, parental SES, and subjects and items as random effects. The results showed a significant main effect of condition [F(2, 355.68) = 46.44, *p* < 0.001, η^2^ = 0.21], but no main effect of group, and no interaction between group and condition. Further analyses confirmed that both groups showed the same pattern on differences across the three unattended conditions, with the unattended acoustic interference (MuR) showing more encoding and higher reconstruction accuracy than the two unattended linguistics distractors (English, Latin). For monolinguals, the results of the pairwise t-tests were t = 7.64, *p* < 0.001, *d* = 0.22 for English–MuR vs English–English comparison, and t = 9.85, *p* < 0.001, *d* = 0.29 for English–MuR vs English–Latin comparison. For bilinguals they were t = 5.55, *p* < 0.001, *d* = 0.16 and t = 6.62, *p* < 0.001, *d* = 0.18 respectively. Neither group showed a significant difference in reconstruction accuracy for the unattended envelopes between the English–English and English–Latin conditions. Finally, the between group comparisons showed a significantly higher encoding of unattended envelopes in the English–MuR condition for monolinguals compared to bilinguals [t = 4.05, *p* < 0.001, *d* = 0.11]. These results are summarised in Fig. 3b.

In sum, our results revealed that monolingual children modulate the accuracy of attended stimulus reconstruction as a function of the type of interference, with linguistic distractors (English, Latin) most strongly interfering with the reconstruction of the attended stream. In contrast, bilingual children showed weaker differentiation in the encoding of attended speech across conditions. The key factor driving these between-group differences appears to be the strength of encoding in conditions of little or no interference (Single talker, English–MuR), with significantly stronger encoding in monolinguals than in bilinguals here. Monolingual and bilingual children showed comparable patterns of reconstruction accuracy of unattended speech.

## Discussion

Building on the substantial evidence that learning and using multiple languages modulates selective attention in children^61^, the current experiment investigated the mechanisms that drive this modification. Using a dichotic listening task we assessed the patterns of responses to different types of interference in monolingual and bilingual children aged 7–12; comparing their behavioural comprehension scores and their cortical tracking of attended and unattended speech envelopes. Despite equivalent behavioural performance, we saw clear differences in the way monolinguals and bilinguals encoded attended speech, confirming that the processing demands of bilingualism shape the supporting neurocognitive architecture^32^. Most importantly however we observed that, instead of enhanced attentional capacity, these neuroadaptive modifications appear to reflect its redistribution, arguably aimed at economising the available resources to support optimal behavioural performance. We discuss these results in more detail below.

In terms of behavioural comprehension scores, our results clearly showed that all children performed the task equally well, and were able to process the attended stories for meaning. This aligns with the general pattern observed in dichotic listening studies that the information presented to the attended ear can usually be processed with very few errors^51,62^. Importantly however, data showed no difference in the pattern of comprehension scores between monolingual and bilingual children, with both groups achieving high comprehension scores across the board, but finding the English–English condition most difficult. Similar to the arguments already made in the literature^24^, this finding that both groups achieved equivalent high-level performance can be taken to imply that any modification to the underlying neural mechanisms in the bilingual group could be considered as adaptation aimed at supporting such performance, made necessary by the increased processing demands of the bilingual environment.

The analysis of the neural data focused on reconstruction accuracy of attended and unattended speech envelopes from the EEG data as the index of attentional encoding. As reviewed in the Introduction, it has been well established in both children and adults that cortical activity encodes the temporal envelope of speech, synchronizing to its slow amplitude modulations^63,64^. Selective attention robustly influences these synchronizations, with the results showing preferential tracking of the attended stream over the ignored one^65,66^. These synchronizations between the auditory signal and the neural data were typically investigated by assessing their linear relationship using cross-correlation or forward modelling; here we used a backward ‘stimulus reconstruction’ approach that has been gaining increased popularity in the recent literature^41,44,56,67^ as it offers advantages such as providing increased sensitivity to signal differences between highly correlated EEG channels^43^.

Consistent with the existing evidence^65,68,69^ our results showed a robust effect of attention, with higher reconstruction accuracy scores consistently seen for the attended than for the unattended envelopes in both groups. Given that reconstruction scores reflect how much stimulus-relevant information is encoded in the EEG signal and how well we can model this, these results imply that attended streams were encoded more strongly than the unattended streams. Also consistent with the existing data^39,53,70^ we saw that the type of interference influenced attentional processing; with linguistic distractors (English and Latin) reducing reconstruction accuracy of the attended envelopes more strongly than the less interfering distractors (Single talker and English–MuR conditions). This is arguably because attentional selection between competing streams of information can be achieved either on the basis of lower-level sensory differences between them, or based on higher-level syntactic and semantic information—with the latter argued to occur later and require more processing capacity^30,71^. The separation between the two streams in the linguistic distractor conditions is more likely to require this latter type of processing, more robustly impacting on the attentional capacity available for the processing of attended stream in these conditions. Alternatively, this pattern of results might be explained in terms of increased difficulty of auditory object formation and selection in the linguistic distractor conditions^72^, where the similarity between the attended and the unattended streams might cause them to be perceived as a unified auditory object, thus resulting in poorer sensitivity to the content of the attended target stream.

The key finding of our study however was that the attentional encoding across conditions differed between the monolingual and the bilingual children. In the monolingual group, we saw a prominent contrast between the conditions with low or no interference and the linguistic interference conditions; yet this effect was markedly attenuated in the bilingual group (Fig. 3). The differential patterns of encoding in monolingual and bilingual listeners observed here replicates the results found in adults^27^, adding further support to the hypothesis that bilingualism modifies the neural mechanisms of selective attention across the lifespan^14,73,74^. In the Introduction, we presented two accounts that might explain the possible mechanisms of this modification. The first was that the need for constant management and inhibition of competing languages in bilinguals enhances their capacity for selective attention, resulting in better performance and increased attentional control^10^. The second was that these demands of selection and inhibition will themselves utilise some of the existing attentional resources, which might impact on the available attentional capacity and require that the remaining resources are optimised in order to achieve full task performance. Our results showed no evidence for the enhanced attentional capacity, behaviourally or neurally, in the bilingual group. In contrast there was a trend for weaker neural encoding in bilinguals overall (r_attd_ = 0.054 for bilinguals vs r_attd_ = 0.066 for monolinguals), and significantly weaker reconstruction in conditions of low or no interference in bilingual compared to monolingual children, lending support to the second proposition.

The observed indication of reduced cortical encoding overall in bilinguals is not without a precedent, with examples of reduced neural activity during selective attention tasks most commonly seen in the cortical areas associated with conflict processing. For instance, functional imaging during a Flanker task performed by bilinguals and monolinguals^75^ revealed significantly lower patterns of activation in the anterior cingulate cortex (ACC) for bilinguals, leading the authors to conclude that ‘bilinguals…resolve cognitive conflicts with less neural resource’. A similar fMRI study of a Stroop-like switching task^74^ also found that monolinguals activated the ACC during the task, whereas bilinguals did not. An ERP study tracking bilingual and monolinguals’ neural responses during a variety of selective attention tasks^26^, predicted superior performance (greater accuracy and faster reaction times) and larger N2 amplitudes for bilinguals relative to monolinguals. On the contrary, behaviour was equivalent between the two groups; and the monolingual group exhibited larger N2 amplitudes than the bilingual group during the Stroop task. The Simon task also elicited the ‘unexpected and surprising’ result that monolinguals demonstrated larger P3 amplitudes than bilinguals. Furthermore, higher ERN amplitudes for bilinguals than monolinguals in the final Flanker task, which would usually be interpreted as evidence of enhanced cognitive control, were due to a longer tail for incongruent trials, indicating a prolonged post-response conflict and slower recovery for bilinguals in these trials. Taken together, this evidence supports the hypothesis that different configurations of the underlying neurofunctional architecture can support equivalent behavioural performance, with these different configurations reflecting different processing demands presented to the system over time. This functional plasticity (also known as degeneracy in the scientific literature^76–78^) is a common feature in biological systems, allowing flexible adaptation to changing environments. Hence, while our findings reveal that the management of competing languages draws on attentional resources in bilingual children, they do not show any adverse effects on performance—the outcome is primarily indicative of the modifications to the underlying processing networks that are aimed at supporting performance. In fact, as mentioned in the Introduction, these results could be interpreted as showing increased flexibility in the usage of the available resources in bilingual children, enabling them to do ‘more with less’.

We next turn to the more specific pattern of reduced cortical tracking of the attended speech envelope in bilinguals observed in our study, where this was most prominent in the Single talker and English–MuR conditions—the two conditions with weakest interference, and thus requiring least effort to comprehend the attended steam. We hypothesise that this directly results from the need to economise the available attentional capacity in order to support optimal behavioural performance. To understand this, it is again necessary to recall that behavioural comprehension scores were equivalent between the groups for all conditions. Yet, achieving optimal behavioural performance is not equally demanding across different conditions, and can arguably be more easily accomplished with reduced attentional resources in the conditions that are less taxing for the processing system. We therefore assume that this reduction in cortical tracking in the conditions of weak or no interference in bilinguals arises because it can be most easily accommodated while still retaining full behavioural performance. In contrast, reductions of attentional encoding in conditions with stronger interference (English–English and English–Latin) would likely lead to diminished performance compared to the monolingual group. Whilst tentative, this interpretation aligns with evidence from research into the mechanisms of adaptive neural plasticity, which suggest that ‘experiences contributing to mastery over environmental challenges modulate neural responses in ways that enhance optimal performance’^79^.

The final set of findings to address concerns the pattern of reconstruction accuracy scores seen for the unattended streams. Here we saw that, in both groups, the unattended MuR stream was significantly better reconstructed than the unattended Latin and English stories. In addition, the MuR encoding was stronger in the monolingual than in the bilingual group. Both of these findings might be explained by the same mechanisms discussed above, with the selection between competing streams being less demanding for the MuR distractor and for monolinguals, thus impacting least on processing capacity available for encoding. However, it is more likely that the strong MuR encoding reflects the fact that the unattended MuR envelopes used in the experiment were generated from the same narratives that the participants were presented with as target stories in their attended ear. Given that the MuR envelope largely preserves the spatio-temporal features of the source utterance, it is unsurprising that there is a high degree of similarity between the envelope reconstruction scores for attended and unattended steams in the English–MuR condition. Despite this, our results showed that the attended steam was more strongly encoded than the unattended steams (significantly so in the monolingual group), adding further evidence that attention significantly influences the neural encoding of speech envelope^69^.

In sum, the current study investigated the mechanisms underlying the modification of selective attention in bilingual children. The data showed no evidence for the enhanced attentional capacity in the bilingual group. Instead, we observed equivalent behavioural performance, coupled with a modified pattern of neural encoding that was most prominent in conditions of weak or no interference. We interpret this data as showing that the available resources are economised to support optimal behavioural performance; potentially suggesting increased flexibility of their usage in response to the demands of bilingual language processing. Overall however, these results emphasise that the demands of learning and using multiple languages modify the mechanisms of selective attention in children, which may have significant consequences for their academic performance and beyond.

## Data Availability

The datasets generated and analysed in the current study are available on request from the first author.
